# Comparison of the Piezoelectric Response of Zwitterionic Liquids and Their Ionic Liquid Counterparts

**DOI:** 10.1021/acs.jpcb.6c05681

**Published:** 2026-10-24

**Authors:** Andrew J. Wendel, Angira Roy, Sheryl S. Blanchard, Gary A. Baker, G. J. Blanchard

**Affiliations:** † 3078 Michigan State University, Department of Chemistry, East Lansing, Michigan 48824, United States; ‡ University of Missouri—Columbia, Department of Chemistry, Columbia, Missouri 65211, United States

## Abstract

We have evaluated the direct piezoelectric response of four zwitterionic liquids (ZwILs): 4-(1-(2-(2-methoxyethoxy)­ethyl)-1*H*-imidazol-3-ium-3-yl)­butane-1-sulfonate ((eo)_2_Im^+^(CH_2_)_4_SO_3_^–^), 3-(1-(2-(2-methoxyethoxy)­ethyl)-1*H*-imidazol-3-ium-3-yl)­propane-1-sulfonate ((eo)_2_Im^+^(CH_2_)_3_SO_3_^–^), 3-(1-(2-(2-methoxyethoxy)­ethyl)-1*H*-imidazol-3-ium-3-yl)­propane-1-sulfonate ((eo)_3_Im^+^(CH_2_)_3_SO_3_^–^), and 3-(1-(2,5,8,11,14,17,20-heptaoxadocosan-22-yl)-1*H*-imidazol-3-ium-3-yl)­propane-1-sulfonate ((eo)_7_Im^+^(CH_2_)_3_SO_3_^–^) in both zwitterionic and ionic forms; the ionic forms were generated by protonating the ZwILs with trifluoromethanesulfonic acid (triflic acid, TA). The data show that the direct piezoelectric responses of the zwitterionic forms are small relative to the response of the reference ionic liquid, 1-butylpyridin-1-ium bis­((trifluoromethyl)­sulfonyl)­imide (C_4_Py TFSI), and that the ionic (protonated) forms of the ZwILs exhibit a piezoelectric response that depends on the extent of protonation. We relate these results to the structure-dependent dipolar properties of the species and to how these properties influence the pressure-induced crystallization process required to manifest the direct piezoelectric effect in liquids.

## Graphical abstract

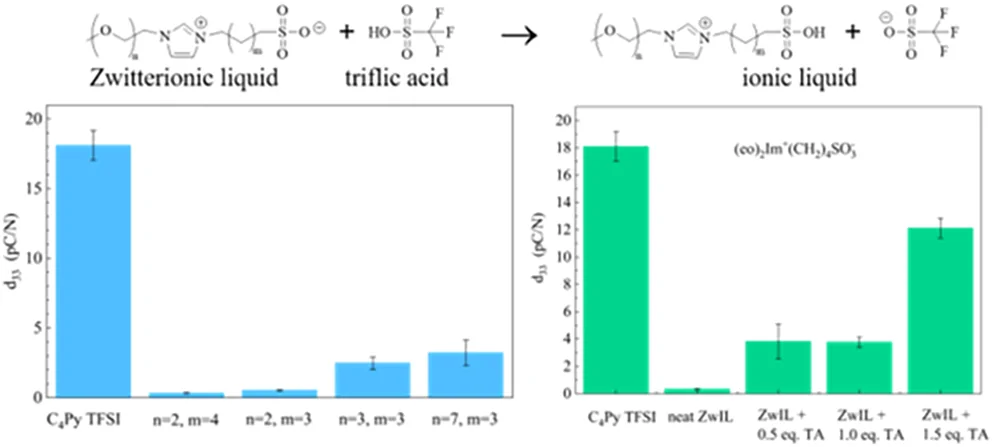

## Introduction

Ionic liquids (ILs) and deep eutectic solvents (DESs) are two related families of compounds that have found extensive application because they exhibit bulk properties that are favorable for numerous applications, and these systems afford compositional and structural control over multiple properties of interest.^1–6^ The discovery of the direct piezoelectric response in ILs and, more recently, in DESs has driven a search to understand the dominant structural and physical factors that contribute to this novel effect.^7–10^ The ability to extract a piezoelectric response from a liquid-phase material is attractive for sensing applications in which solid-state piezoelectric materials are of limited utility, as well as for novel applications such as piezo-pneumatics. Because this is an emerging area, the piezoelectric responses of ILs and DESs are typically smaller in magnitude than the best solid-state materials, such as Pb­[Zr_*x*_Ti_1–*x*_]­O_3_ (0 ≤ *x* ≤ 1) (PZT), and an important question is how to optimize the piezoelectric response in liquid-phase materials.

Subsequent to the initial discovery of the direct piezoelectric effect in ILs, the mechanism was determined to be the pressure-induced crystallization of the liquid-phase material, in which the pressure-induced crystal is the piezoactive material.^11^ The piezoelectric response in both ILs and DESs thus depends on the propensity of the liquid to undergo a pressure-induced phase transition to a (noncentrosymmetric) crystal, and understanding the factors that optimize both the propensity to form crystals and the kinetics of crystal formation is an important area of investigation. To that end, it is useful to consider the potential utility of zwitterionic systems for piezoelectric applications.

The appeal of zwitterionic species for this work lies in their characteristically large permanent dipole moments. This property stems from the structurally enforced charge separation within the molecule, and many zwitterionic species can exhibit relatively large dipole moments compared with more traditional systems with dissociable cationic and anionic species. While the large dipole moments characteristic of ZwILs may appear favorable for crystallization, strong dipole–dipole interactions do not necessarily favor a piezoelectric response because they can promote antiparallel alignment of dipolar species (dipolar stacking), which can lead to the formation of nominally centrosymmetric structures. Resolving the relative importance of these opposing factors is a goal of the work reported here. Recent molecular dynamics (MD) simulations of three members of the same propanesulfonate ZwIL series likewise found large molecular dipoles and frequent antiparallel dipole arrangements, supporting this structural picture.^12^

We have chosen a series of ZwILs to evaluate their piezoelectric response as a function of zwitterionic structure (Figure 1). These ZwIL structures allow for a direct comparison with their corresponding IL structures by means of protonation (Figure 2). Titration of the ZwILs with trifluoromethanesulfonic acid (triflic acid, TA) protonates the sulfonate functionality, converting the ZwIL to a cationic species with trifluoromethanesulfonate (triflate) as the counteranion. The protonation of the ZwIL is expected to be quantitative given the large difference in p*K*_a_ values for alkylsulfonic acids (*ca*. +2) and TA *(ca*. −14.7).^13^ Measurement of the direct piezoelectric response of the neat ZwILs and after addition of 0.5, 1.0, and 1.5 equiv of TA reveals significant, system-dependent variation in the piezoelectric response that depends on both the identity of the ZwIL and the amount of TA added. We understand these results in the context of system-specific changes in the transient pressure-induced crystallization process.

**Figure 1:**
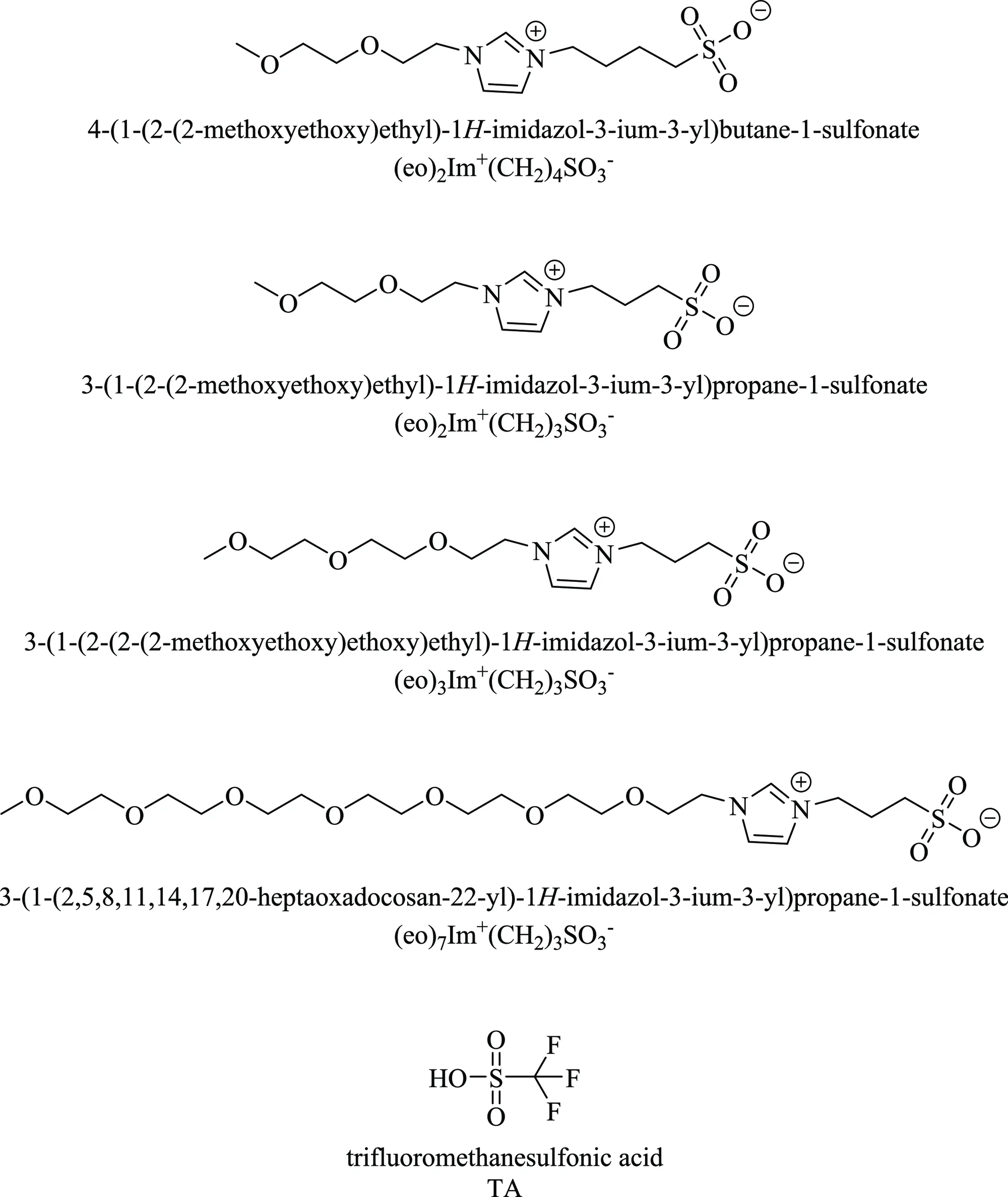
Chemical structures of the compounds used in this study.

**Figure 2:**
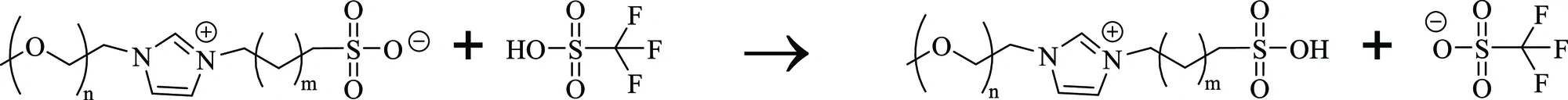
Schematic illustration of zwitterionic liquid (ZwIL) titration with triflic acid (TA) to generate the corresponding ionic liquid (IL).

## Materials and Methods

### Chemicals

The zwitterionic liquids used in this work are 4-(1-(2-(2-methoxyethoxy)­ethyl)-1*H*-imidazol-3-ium-3-yl)­butane-1-sulfonate ((eo)_2_Im^+^(CH_2_)_4_SO_3_^–^), 3-(1-(2-(2-methoxyethoxy)­ethyl)-1*H*-imidazol-3-ium-3-yl)­propane-1-sulfonate ((eo)_2_Im^+^(CH_2_)_3_SO_3_^–^), 3-(1-(2-(2-methoxyethoxy)­ethyl)-1*H*-imidazol-3-ium-3-yl)­propane-1-sulfonate ((eo)_3_Im^+^(CH_2_)_3_SO_3_^–^), and 3-(1-(2,5,8,11,14,17,20-heptaoxadocosan-22-yl)-1*H*-imidazol-3-ium-3-yl)­propane-1-sulfonate ((eo)_7_Im^+^(CH_2_)_3_SO_3_^–^). The experimental procedures for the preparation, purification, and characterization of the ZwILs followed previously reported synthetic strategies.^12^ Briefly, the desired monomethyl polyether (*e.g*., diethylene glycol methyl ether for (eo)_2_Im­(CH_2_)_3_S) was dissolved in a 1:1 (v/v) mixture of THF and water and deprotonated with sodium hydroxide in an ice bath prior to the slow, dropwise addition of *p*-toluenesulfonyl chloride (TsCl). Following reaction, extraction with DCM, drying over magnesium sulfate, and solvent removal by rotary evaporation afforded the monotosylated polyether. The monotosylated polyether was then reacted with imidazole and NaOH under reflux in THF for 24 h. After cooling and solvent removal, the DCM-extracted fraction was dried and concentrated by rotary evaporation to afford the corresponding imidazole-functionalized polyether as a colorless liquid in ∼90% yield. The imidazole-polyether was subsequently dissolved in acetonitrile and quaternized under an argon atmosphere by reaction with a cyclic sulfonate ester (*e.g*., 1,3-propane sultone for (eo)_2_Im­(CH_2_)_3_S), thereby introducing the negatively charged sulfonate terminus. Workup with diethyl ether afforded the desired ZwIL. Trifluoromethanesulfonic acid (Sigma-Aldrich, >99%) was used as received and was transferred by syringe between vessels sealed with septa. The ionic liquid 1-butylpyridin-1-ium bis­((trifluoromethyl)­sulfonyl)­imide (C_4_Py TFSI, Sigma-Aldrich) was purified prior to use by an established protocol.^14,15^

### Piezoelectric Response Measurements

The instrument used to acquire direct piezoelectric response data from the samples has been described in detail elsewhere,^16^ and we provide a brief recap here. The instrument was designed and built in-house. The sample holder is a cylinder-and-piston assembly in which the cylinder is made of steel and the piston is made of Delrin; the seal between the cylinder and piston is formed using a Buna-N O-ring. The apparatus used to apply pressure to the samples is a second-class lever system, with the applied force varied by changing the lever-arm length. The signal of interest consists of current transients that result from the application of force to the samples. The current transients are measured using an electrometer (Keithley 6517A). Electrometer control and data acquisition are accomplished with a LabVIEW virtual instrument (VI) developed in-house. The force applied to the lever was quantified using a calibrated digital force gauge (Nidec model FG-3009). Each determination of the current vs force data (Figures S1–S5) is based on 36 individual measurements for a given system, and the instrument is calibrated daily by the acquisition of current vs force data for C_4_Py TFSI.

### Semiempirical Calculations

Semiempirical computations were performed with HyperChem 8.0 using the PM3 parametrization.

## Results and Discussion

There is a large literature on ILs, with much fundamental information and a wide variety of applications.^17–32^ In contrast, the literature on ZwILs is much more limited, with some focus on the dielectric response of these materials.^12,33–47^ Despite the large permanent dipole moments characteristic of zwitterions, relatively few ZwILs have been reported, and structural features of known ZwILs, such as pendent poly­(ethylene oxide) chains, can hinder efficient packing and thereby suppress solidification at room temperature.^36^ These structural properties, which are synthetically tunable, may provide an opportunity to create a system poised to undergo a pressure-induced liquid-to-solid phase transition into a crystalline state.

The poly­(ethylene oxide)-imidazolium-alkylsulfonate ZwILs afford both an opportunity to understand the roles of poly­(ethylene oxide) and alkylsulfonate pendent moieties in determining the piezoelectric response and a direct means of comparing ZwIL and IL versions of these systems. We compare the direct piezoelectric response of the neat ZwILs in Figure 3, where the *d*_33_ value for the ionic liquid C_4_Py TFSI is provided as a reference.^16^

**Figure 3:**
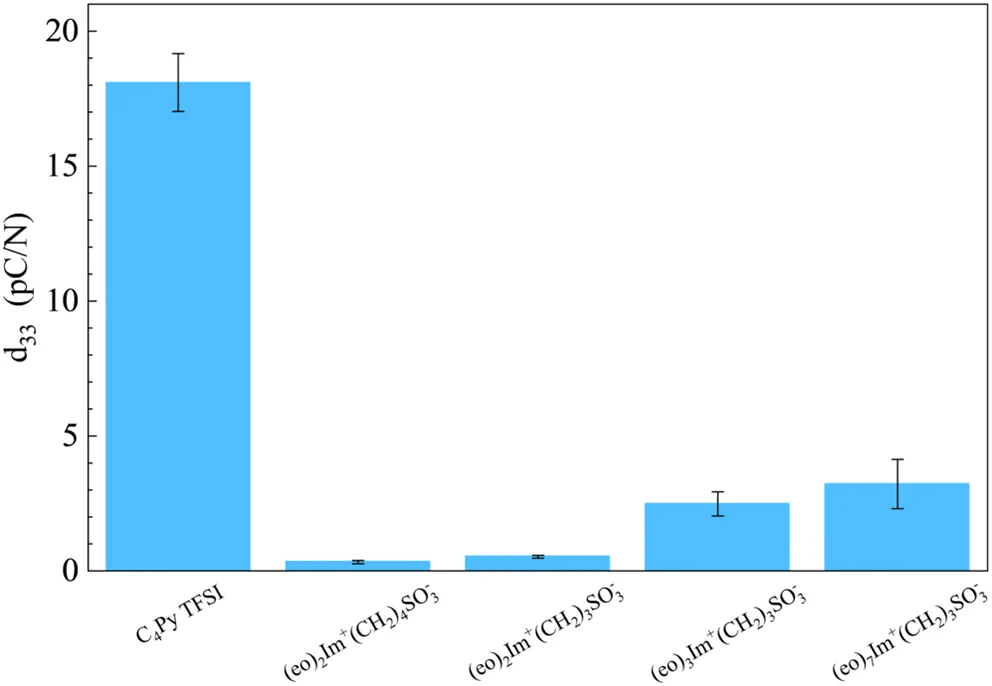
Experimental *d*_33_ data for the ZwILs indicated. The *d*_33_ value, which describes the amount of charge generated per unit of force, for C_4_Py TFSI is included as a reference. Uncertainties are standard deviations of the least-squares best fit slopes of the current vs force data.

There are several interesting features contained in these data. First, for all the ZwILs, the piezoelectric coefficient, *d*_33_, is much smaller than that of C_4_Py TFSI, a pyridinium-based IL. This finding can be rationalized by the tendency of strong dipolar interactions (*vide infra*) to promote antiparallel dipole pairing or stacking. For rigid achiral systems, such arrangements can favor ordered structures containing a center of inversion; centrosymmetric structures are piezoelectrically inactive. Second, *d*_33_ for the ZwILs increases with increasing ethylene oxide chain length. Increasing chain length is expected to increase conformational freedom and the population of ethylene oxide *gauche* conformers, which can disrupt packing in the pressure-induced ZwIL solid and thereby reduce the fraction of ZwIL crystalline structures possessing a center of inversion. Of course, although the number of *gauche* defects increases with chain length, their fractional population depends strongly on factors such as packing density and temperature. Individual *gauche* conformations remain slightly less stable than *anti* conformations, but longer chains have a greater statistical likelihood of containing one or more gauche defects. At the same time, recent MD simulations of the corresponding propanesulfonate ZwILs show that longer poly­(ethylene oxide) chains can enhance nanoscale segregation and intermediate-range order, particularly for the longest chain, indicating that the chain-length dependence of *d*_33_ is unlikely to reflect conformational disorder alone.^12^

With this framework, we consider the effect of adding triflic acid to the neat ZwILs (Figure 2). As noted above, TA protonates the sulfonate group of the ZwIL, converting the ZwIL into a cation with triflate as the counteranion because of the large difference in p*K*_a_ values of the two acids. Figure 4 shows these data for each ZwIL. For each ZwIL system, the first bar in each panel is C_4_Py TFSI, included as a reference. The second bar corresponds to the neat ZwIL, whereas the third, fourth, and fifth bars correspond to additions of 0.5, 1.0, and 1.5 equiv of TA, respectively. Addition of 0.5 equiv of TA yields a nominal 50:50 mixture of ZwIL and IL; 1.0 equiv yields nominally 100% IL; and 1.5 equiv yields IL with 0.5 equiv excess TA. No consistent trend is observed in the titrations of the four ZwILs. This is not a surprising result in light of the operative mechanism of the piezoelectric response, which is the formation of a crystalline solid species upon the application of pressure.^11^ The structural details of the transient crystalline species for each system are not resolved at this point, but different mixtures of ZwILs and ILs are expected to yield crystalline species that are not directly comparable to one another. There is experimental evidence in the piezoelectric response data (Figures S1–S5) that underscores the differences in crystalline systems formed under pressure. Specifically, the sign of the current transients varies with ZwIL identity and system composition. We summarize the signs of the current transients for each system in Table 1. The sign of the current transient is determined by the orientation of the crystals formed in the sample cell upon the application of pressure. The transient crystals will form either on the face of the cylinder or on the head of the piston. The orientation of the crystal, controlled by the interactions of the piezo-active material with the surface, determines the sign of the current transient. Changing the crystal structure by changing its constituent species can alter the crystal orientation, and the information in Table 1 demonstrates changes in sign for a given ZwIL with the addition of TA.

**Figure 4:**
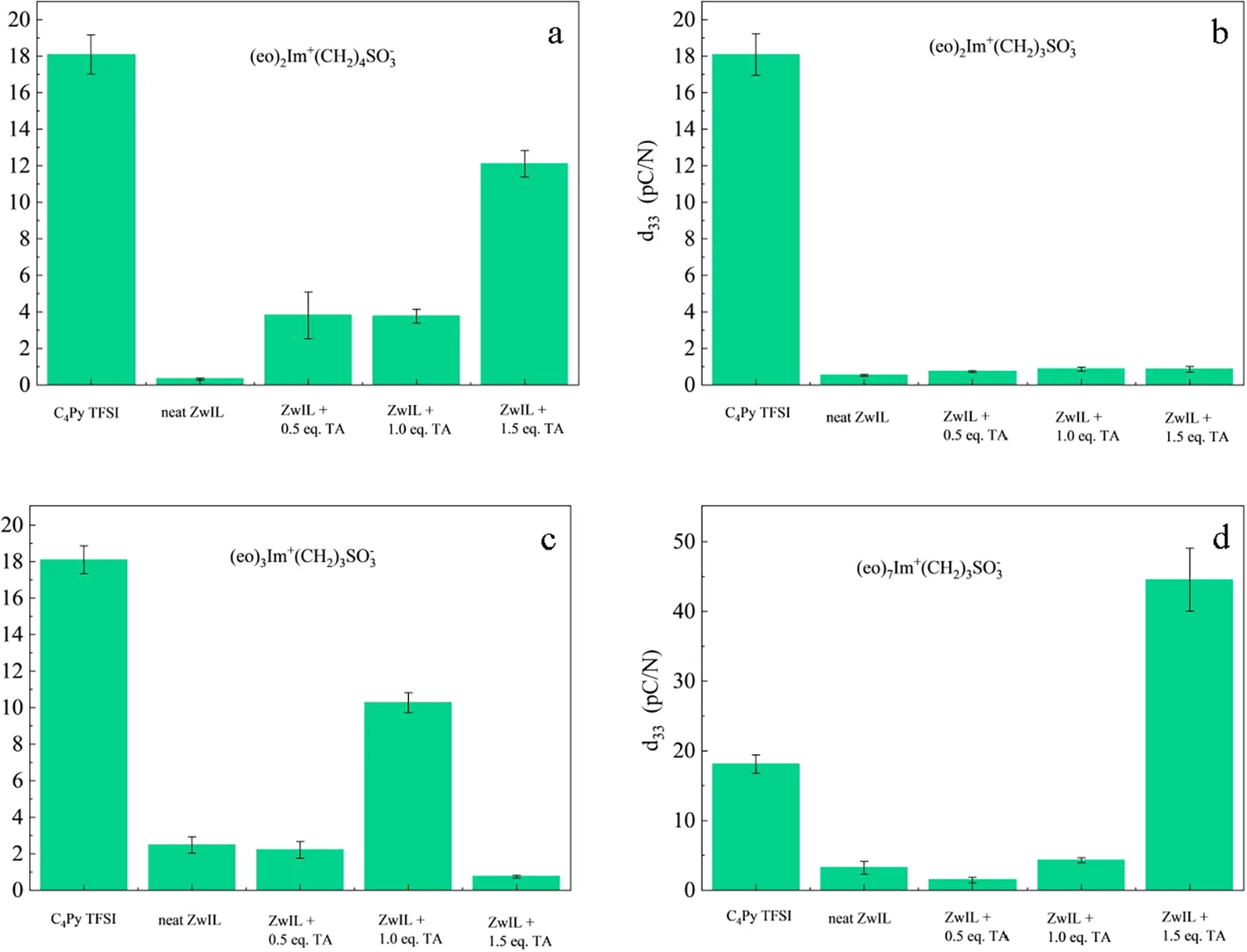
Piezoelectric coefficient *d*_33_ as a function of triflic acid (TA) addition for (a) (eo)_2_Im^+^(CH_2_)_4_SO_3_^–^, (b) (eo)_2_Im^+^(CH_2_)_3_SO_3_^–^, (c) (eo)_3_Im^+^(CH_2_)_3_SO_3_^–^, and (d) (eo)_7_Im^+^(CH_2_)_3_SO_3_^–^. The *d*_33_ value for the IL C_4_Py TFSI is included in each panel as a reference. Uncertainties are standard deviations of the least-squares best fit slopes of the current vs force data.

**Table 1:** Sign of the Pressure-Induced Current Transients for ZwILs as a Function of Triflic Acid (TA) Addition

System	Neat ZwIL	ZwIL + 0.5 equiv TA	ZwIL + 1.0 equiv TA	ZwIL + 1.5 equiv TA
(eo)_2_Im^+^(CH_2_)_4_SO_3_^–^	–	–	+	+
(eo)_2_Im^+^(CH_2_)_3_SO_3_^–^	–	+	+	+
(eo)_3_Im^+^(CH_2_)_3_SO^–^	+	+	+	+
(eo)_7_Im^+^(CH_2_)_3_SO_3_^–^	–	–	+	+

While it is not surprising that there are system-dependent changes in transient crystal formation, these data do show, from a phenomenological perspective, that these systems can produce a comparatively large piezoelectric response in some instances (for example, the case of ZwIL + 1.5 equiv TA in Figure 4d), and the chemical and structural factors that contribute to this response require more detailed investigation.

With these findings in mind, it is useful to evaluate the structure-dependent changes in the dipole moments of ZwILs and their corresponding cations because these results underscore the strong dipolar interactions expected for these systems and provide insight into the dependence of the permanent dipole moment on both ethylene oxide and aliphatic chain lengths. For this purpose, we used semiempirical calculations with PM3 parametrization. We recognize that these are relatively low-level calculations and that the actual permanent dipole moments may differ from the computed values. Because the calculated dipole moments are relatively large in all cases, their absolute values are less important for our purposes than the trends in dipole moment magnitude and orientation as a function of molecular structure. With this caveat, we consider the dependence of the permanent dipole moment on the length of the pendent ethylene oxide chain for both the zwitterion and the protonated (cationic) species (Figure 5).

**Figure 5:**
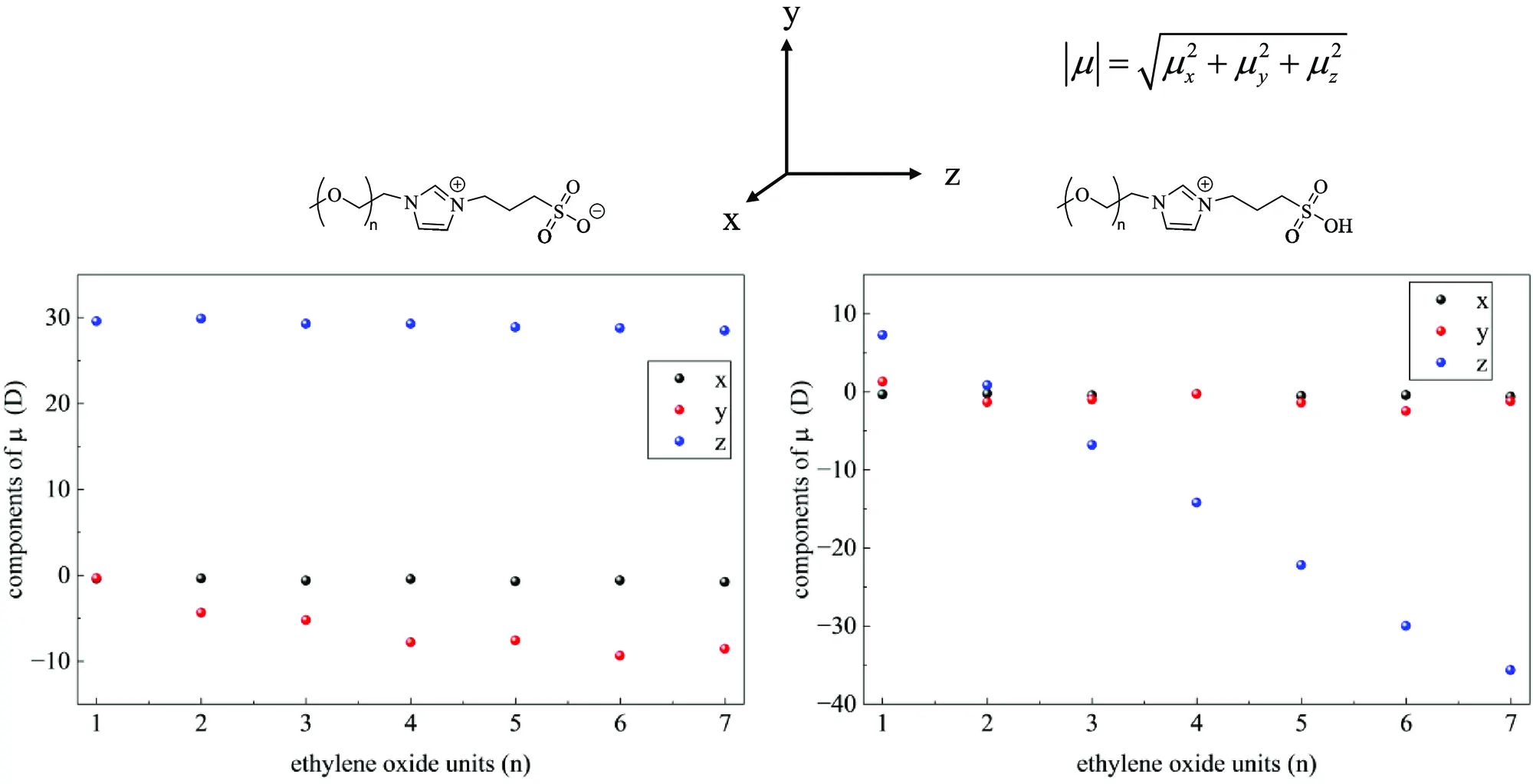
Semiempirical calculations of the Cartesian components of the permanent dipole moment for the zwitterionic (left) and cationic (right) species.

The calculated components of the dipole moment for the zwitterion (Figure 5, left) show that in all cases the dominant dipole moment component lies along the *z*-axis, and the magnitude of the molecular dipole moment remains nearly constant as the number of pendent ethylene oxide units changes. This behavior reflects the fixed charge separation enforced by the zwitterionic structure. The results for the protonated species (Figure 5, right) exhibit a different trend. For all cationic species, the dominant dipole moment component lies along the *z*-axis, but the sign of the *z*-component changes as the number of ethylene oxide units increases, and the magnitude of the permanent dipole moment increases approximately linearly with the number of ethylene oxide units. This behavior arises from the dipole-moment contributions from the imidazolium-propanesulfonic acid group and the imidazolium-ethylene oxide oligomer. These components have nominally opposing orientations and produce a net dipole moment that changes direction with increasing ethylene oxide chain length, reaching a minimum magnitude at *n* = 2. The semiempirical trends therefore suggest that the dipolar contribution from the zwitterionic motif is comparatively insensitive to ethylene oxide chain length, whereas the cationic species exhibit a much stronger chain-length dependence. We recognize that ethylene oxide chain lengths will be characterized by conformational irregularity, which would serve to make the length-dependence of μ_*z*_ shown in the right-hand panel of Figure 5 somewhat less pronounced than the calculations suggest. Recent MD simulations of the corresponding propanesulfonate ZwILs spanning two, three, and seven ethylene oxide units likewise found closely comparable dipole-magnitude distributions and little change in average dipole magnitude from 298 to 700 K, providing qualitative support for this interpretation.^12^ This factor, together with the presence of multiple species in TA-titrated samples, provides a qualitative basis for the system-dependent responses observed here. While not feasible with the present instrumentation, X-ray diffraction measurements of the transient pressure-induced piezoelectric crystals would be particularly valuable for testing this interpretation.

## Conclusions

We have evaluated the direct piezoelectric response of a series of zwitterionic liquids and their corresponding triflate ionic liquids generated by titration of the ZwILs with triflic acid. Our data reveal a small piezoelectric response for the neat ZwILs, likely because strong antiparallel dipolar stacking favors centrosymmetric arrangements during formation of the piezoactive crystalline phase. The addition of TA to the ZwILs does not produce a general trend; instead, the response differs for each ZwIL system examined. We attribute this behavior to the presence of multiple species in these systems and to the ZwIL structure-dependent variation in dipolar contributions to transient piezoelectric crystal formation. Under appropriate conditions, a comparatively large piezoelectric response can be observed, but the structural and physical factors underlying this behavior remain to be elucidated. Future work should therefore examine how intermolecular forces, charge-network organization, and crystallization kinetics govern the formation and orientation of the transient piezoelectric phase.

## Supplementary Material



## Notes

Raw piezoelectric current–time traces for C_4_Py TFSI, neat (eo)_2_Im^+^(CH_2_)_3_SO_3_^–^, and its mixtures with 0.5, 1.0, and 1.5 equiv of triflic acid (PDF)
