# Supplementary material for: Comparison of the Piezoelectric Response of Zwitterionic Liquids and Their Ionic Liquid Counterparts
Source: J Phys Chem B. 2026 Oct 24;130(44):11913–9. doi: 10.1021/acs.jpcb.6c05681 (PMC13746775; doi:10.1021/acs.jpcb.6c05681)
Supplement: Supplementary file 1 [file jp6c05681_si_001.pdf]

Comparison of the Piezoelectric Response of Zwitterionic Liquids and Their Ionic Liquid Counterparts

Andrew J. Wendel,[1] Sheryl S. Blanchard,[1] Angira Roy,[2] Gary A. Baker,[2] and G. J. Blanchard[1,*]

[1] Michigan State University, Department of Chemistry, East Lansing, MI 48824 USA
[2] University of Missouri - Columbia, Department of Chemistry, Columbia, MO 65211 USA

Supporting Information

Figure S1. Current vs. time data for C_4Py TFSI……………………………………………...S2

Figure S2. Current vs. time data for neat $(eo)_2Im^+(CH_2)_3SO_3^-$………………………………….S3

Figure S3. Current vs. time data for $(eo)_2Im^+(CH_2)_3SO_3^-$ with 0.5 equiv CF_3SO_3H……….…..S4

Figure S4. Current vs. time data for $(eo)_2Im^+(CH_2)_3SO_3^-$ with 1.0 equiv CF_3SO_3H……………S5

Figure S5. Current vs. time data for $(eo)_2Im^+(CH_2)_3SO_3^-$ with 1.5 equiv CF_3SO_3H……………S6

* Corresponding author: email: blanchard@chemistry.msu.edu, Tel: +1 517 353 1105

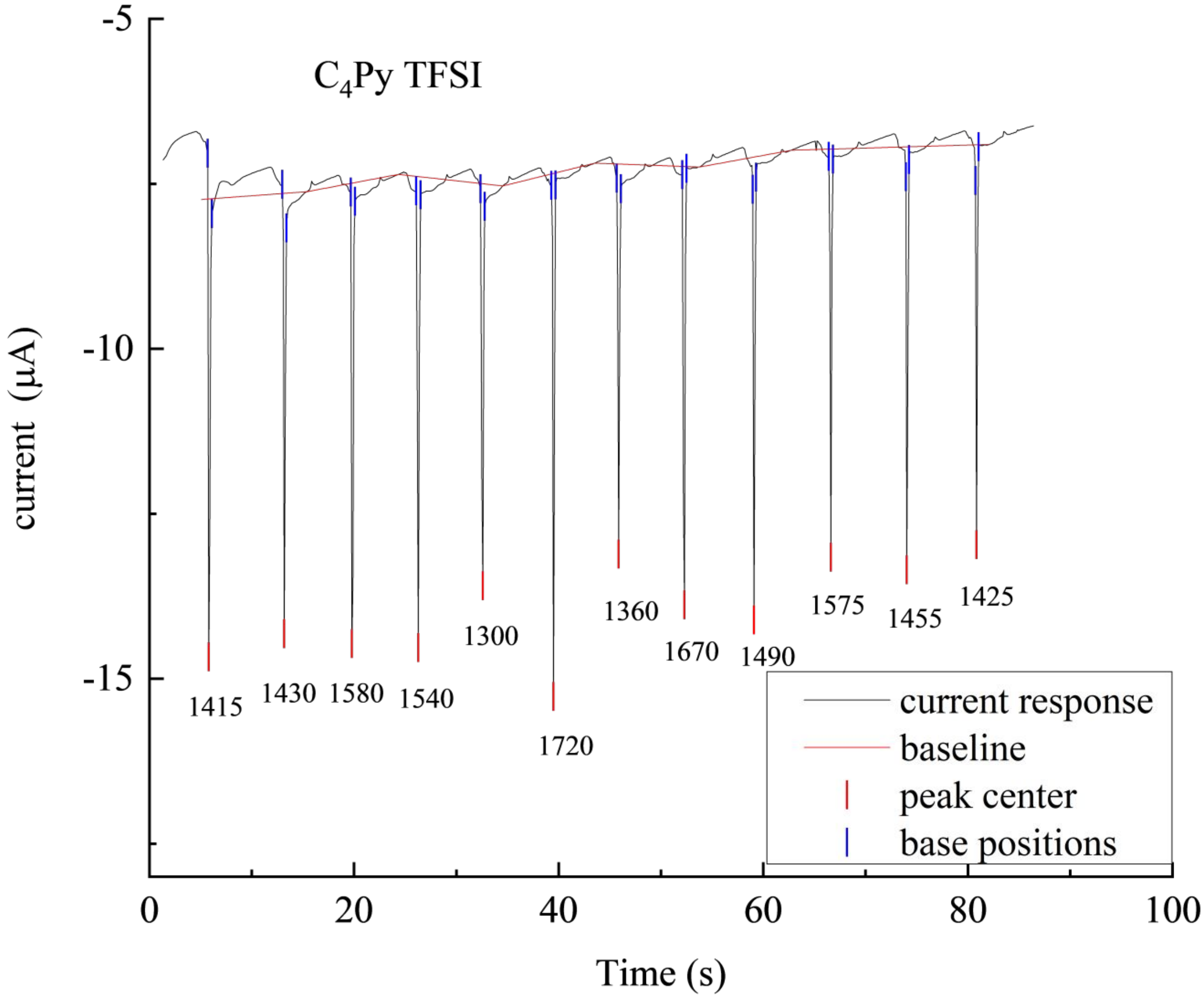

Figure S1. Current vs. time data for C4Py TFSI. Force applied is indicated for each current transient. For the current measurements, baseline, peak maxima and base points are as indicated in the legends.

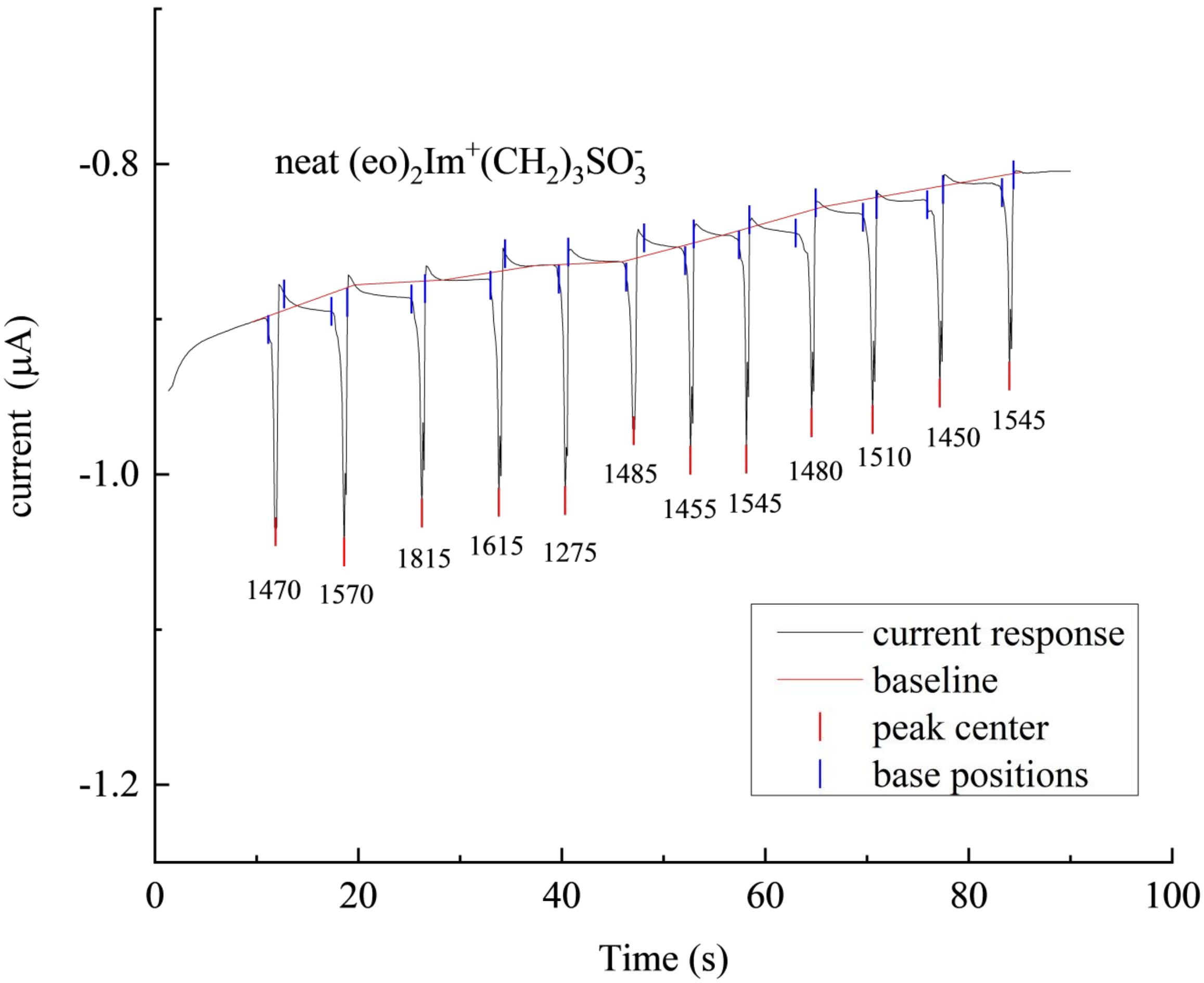

Figure S2. Current vs. time data for neat $(eo)_2Im^+(CH_2)_3SO_3^-$. Force applied is indicated for each current transient. For the current measurements, baseline, peak maxima and base points are as indicated in the legends.

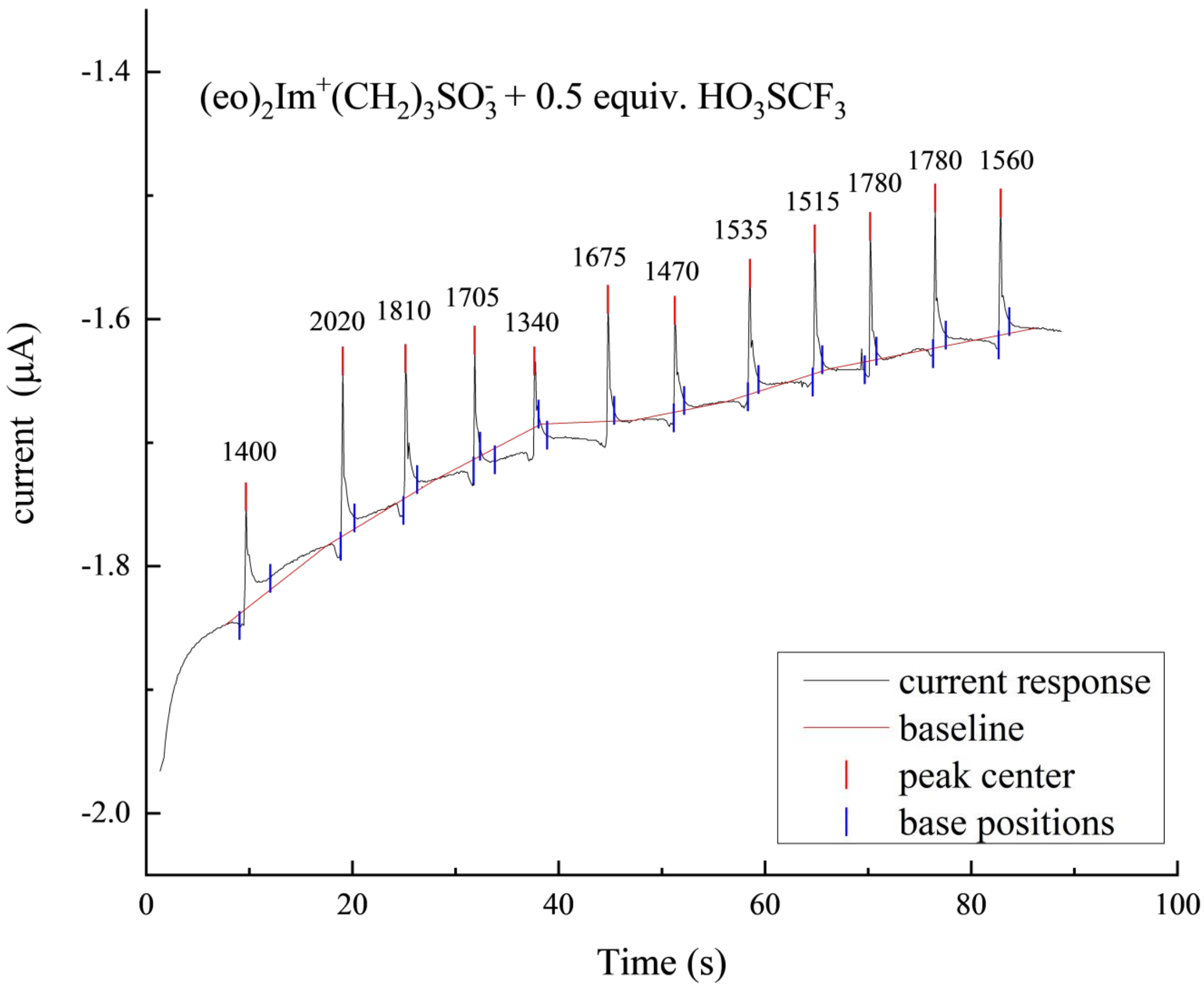

Figure S3. Current vs. time data for $(eo)_2Im^+(CH_2)_3SO_3^-$ with 0.5 equiv CF_3SO_3H added. Force applied is indicated for each current transient. For the current measurements, baseline, peak maxima and base points are as indicated in the legends.

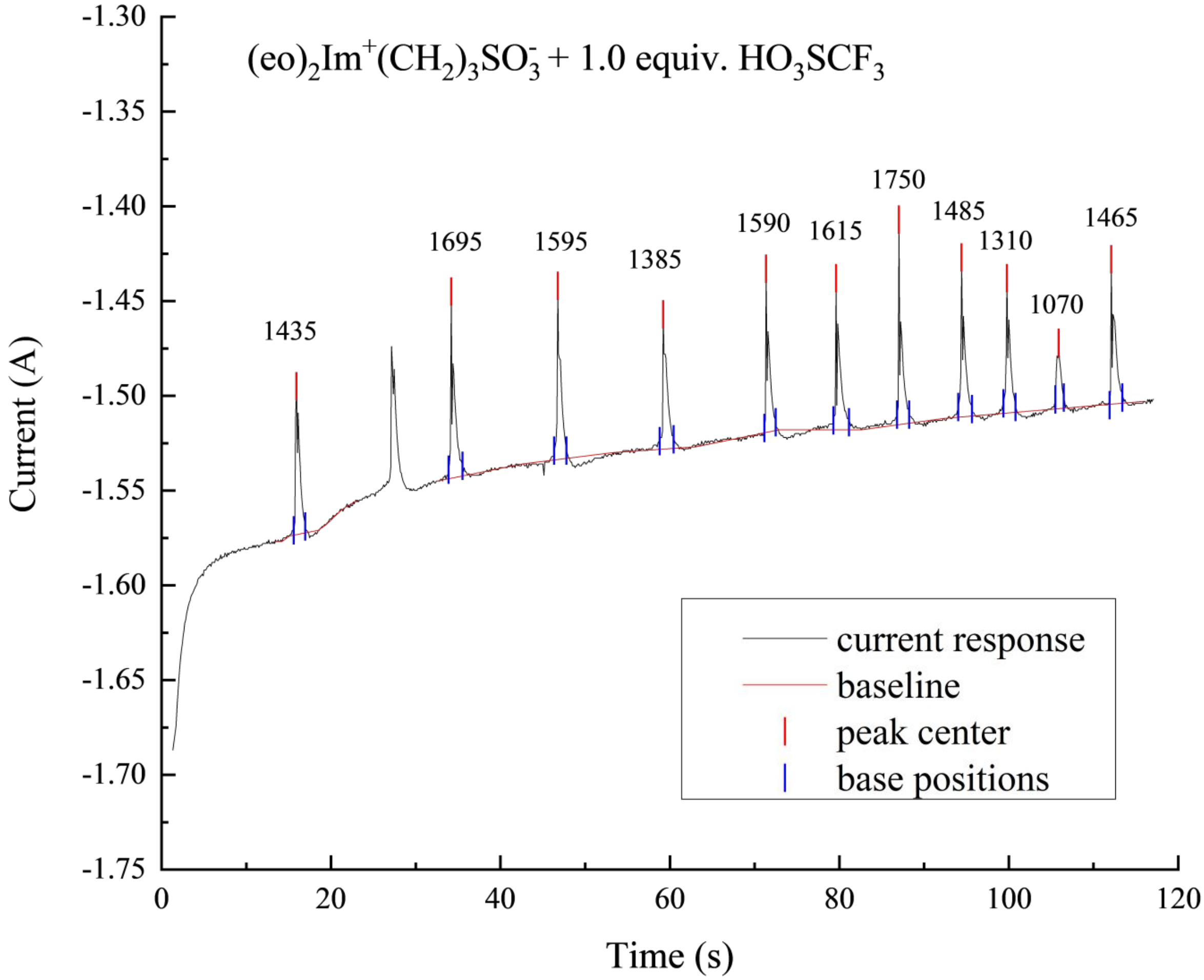

Figure S4. Current vs. time data for $(eo)_2Im^+(CH_2)_3SO_3^-$ with 1.0 equiv CF_3SO_3H added. Force applied is indicated for each current transient. For the current measurements, baseline, peak maxima and base points are as indicated in the legends.

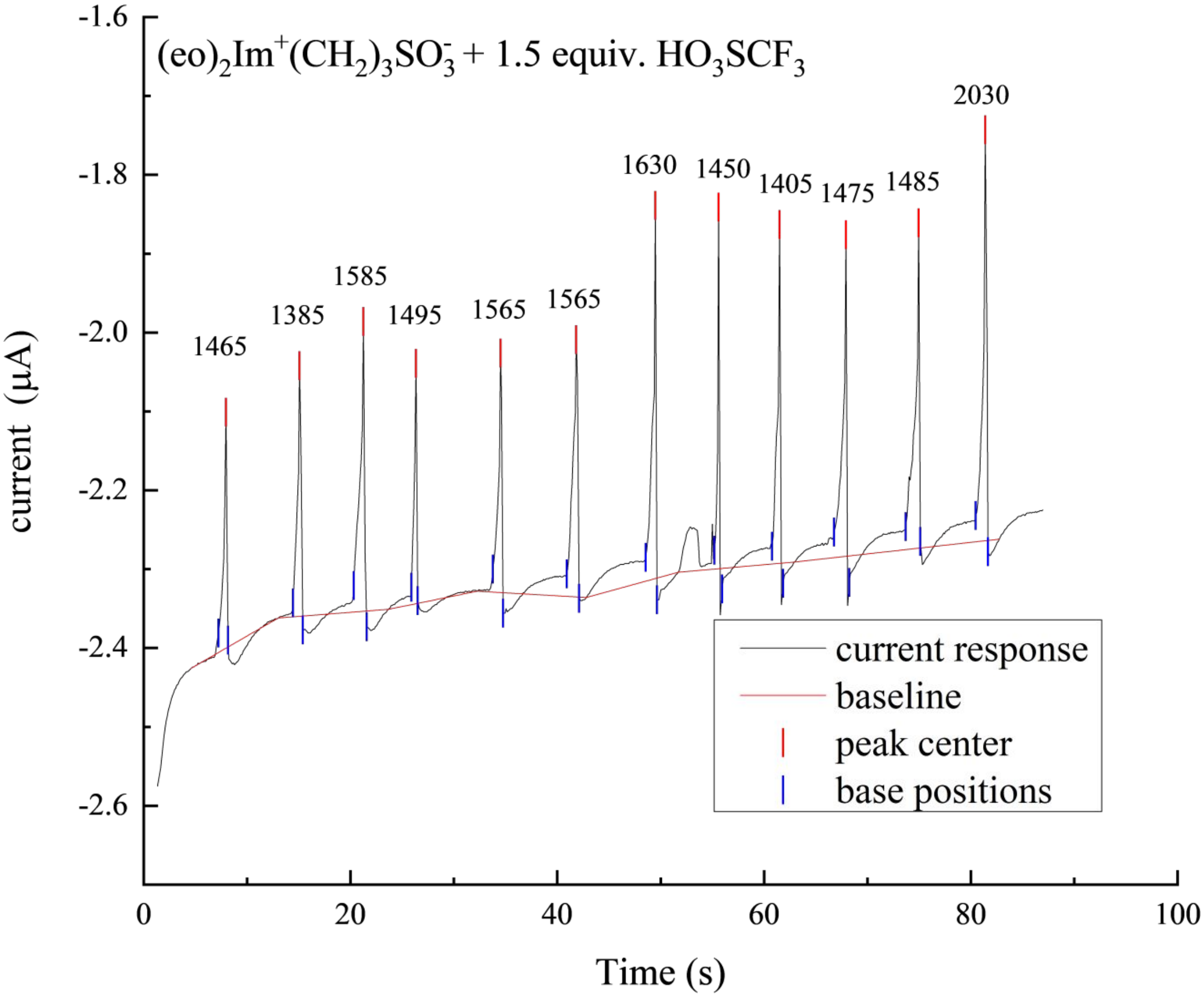

Figure S5. Current vs. time data for $(eo)_2Im^+(CH_2)_3SO_3^-$ with 1.5 equiv CF_3SO_3H added. Force applied is indicated for each current transient. For the current measurements, baseline, peak maxima and base points are as indicated in the legends.
